# Sex differences in motivational biases over instrumental actions

**DOI:** 10.1038/s41539-024-00246-6

**Published:** 2024-10-08

**Authors:** Luigi A. E. Degni, Sara Garofalo, Gianluca Finotti, Francesca Starita, Trevor W. Robbins, Giuseppe di Pellegrino

**Affiliations:** 1 Center for Studies and Research in Cognitive Neuroscience, Department of Psychology, University of Bologna, Cesena, Italy; 2 International School of Advanced Studies, University of Camerino, Camerino, Italy; 3 Department of Psychology, University of Cambridge, Cambridge, UK

**Keywords:** Human behaviour, Classical conditioning

## Abstract

Motivational (i.e., appetitive or aversive) cues can bias value-based decisions by affecting either direction and intensity of instrumental actions. Despite several findings describing important interindividual differences in these biases, whether biological sex can also play a role is still up to debate. By comparing females and males in both appetitive and aversive Pavlovian-to-Instrumental Transfer paradigms we found that, while motivational cues similarly bias the direction of instrumental actions in both sexes, the intensity of such actions is increased by the cue in male participants only. The present results constitute compelling evidence that a crucial motivational bias of daily actions directed to obtaining rewards or avoiding punishments is modulated by biological sex. This evidence sheds new light on the role of sex in motivational processes that underlie decision-making, highlighting the importance of considering sex as a crucial factor in future research on this topic.

## Introduction

Motivational cues are environmental stimuli that have acquired a motivational value through repeated pairing with appetitive (e.g., food) or aversive (e.g., shock) outcomes. Given its numerous implications in clinical contexts as well as daily life^1,2^, a key research area in behavioral neuroscience is the investigation of how such cues can bias value-based decisions^3,4^. Current literature converges on the existence of at least two different types of bias through which motivational cues can alter instrumental actions^5,6^, specifically biases affecting the direction and intensity of actions, each characterized by different functional^7^ as well as neural^8,9^ mechanisms. The direction bias (i.e., outcome-specific transfer) occurs when the outcome-predictive cue affects the choice between two (or more) responses leading to different outcomes, by favoring actions that are congruent with the outcome predicted by the cue (e.g., both cue and action are associated to the same outcome), as compared to incongruent actions (e.g., cue and action are associated to a different outcome). For example, the logo of a particular brand of snacks seen at the supermarket may guide us to buy snacks of that specific brand rather than others. This bias has been related to the use of a cognitive top-down strategy^10,11^, consisting of exploiting the informative value of the cue to search for specific rewards in predictable environments, or to avoid potentially threatening situations. The intensity bias (i.e., general transfer) describes the ability of outcome-predictive cues to generally enhance or energize instrumental responses, even if directed toward different (but motivationally similar) outcomes, as compared to cues associated with no reward (or punishment) or a neutral outcome (i.e., environmental stimuli without any motivational charge). For example, the snack’s logo may drive us promptly toward the nearest food dispenser, independently of the possible snacks that we could find inside. This process is considered to be due to a general, cue-induced increase of motivation toward the outcome^12–14^, e.g., increase in hunger when seeing the snack’s logo, which consequentially increases the intensity (e.g., faster reaction times, stronger grip force) of actions performed to obtain the desired rewarding outcome^15–17^ or avoid the dreaded aversive outcome.

To date, sex differences have been reported for a wide range of cognitive processes^18–21^. Although often no sex differences are reported in decision-making and instrumental actions^22,23^, several authors hypothesize that the absence of differences may conceal variations in the strategy used by males and females to learn and perform these tasks^24,25^. For example, males appear to show more exploration in decision-making tasks than females^25^, who are instead characterized by accelerated action-outcome associations and tend to use systematic strategies for decision-making^24^, even when the optimal way to perform a task would be not to develop early strategies (e.g., Iowa Gambling Task)^22^. Accordingly, differences emerge when looking at implicit aspects such as motivational learning and cue reactivity^23,26–29^, albeit sometimes with mixed results^26,29^. In general, males seem to be characterized by a higher neural and dopaminergic responsiveness to motivational appetitive cues^27,29^ and outcomes^26,28^. In light of this, existing literature has neglected a crucial process in which sex differences might emerge, namely how the motivation acquired by environmental cues is translated into observable biases—of direction or intensity—of instrumental behaviors in humans. Crucially, a deeper understanding of sex differences in such processes may be particularly important to better understand possible differences in the prevalence of and vulnerability to altered motivational processes that can evolve into maladaptive conducts like addiction, depression, anxiety, and gambling^29–31^.

Our study aims to investigate sex differences in how motivational cues can bias the direction and intensity of instrumental actions. To this aim, we used an appetitive Pavlovian-to-Instrumental Transfer paradigm (experiment 1), and re-analyzed the data obtained from a previously published study^15^ with an aversive Pavlovian-to-Instrumental Transfer paradigm (experiment 2) in which both measures of direction (i.e., outcome-specific transfer) and intensity (general transfer) were collected. Given the demonstrated increased responsiveness of males to motivational cues^27,29^ and the hypothesized purely motivational nature of the intensity bias^12–14^, we expect a selective increase in this bias in males. In addition, we want to investigate a possible sex-specific modulation of directionality that reflects higher cognitive abilities instead^10,11^.

## Results

### Experiment 1

#### Explicit measures of liking and wanting

To rule out possible sex differences in reward liking and wanting, we performed two 2 × 3 × 2 mixed-measures ANOVAs with time (pre/post-experiment) and outcome (O_1_/O_2_/O_3_) as within-subjects factors and sex (Males/Females) as between-subjects factor, respectively using the liking and the wanting scores as dependent variables (see Supplementary Materials for descriptive results).

The results of the ANOVA on the liking scores showed no statistically significant main effects of time (*F*_1,39_ = 0.23; *p* = 0.64; BF_10_ = 0.15), outcome (*F*_2,78_ = 0.34; *p* = 0.71; BF_10_ = 0.08), or sex (*F*_1,39_ = 3.69; *p* = 0.062; BF_10_ = 1.4), nor interaction effects (time by outcome interaction: *F*_2,78_ = 0.45; *p* = 0.64; *η*_p_^2^ = 0.01; BF_10_ = 0.08; time by sex interaction: *F*_1,39_ = 0.46; *p* = 0.5; *η*_p_^2^ = 0.01; BF_10_ = 0.21; outcome by sex interaction: *F*_2,78_ = 0.37; *p* = 0.69; *η*_p_^2^ = 0.01; BF_10_ = 0.14; time by outcome by sex interaction: *F*_2,78_ = 0.66; *p* = 0.52; *η*_p_^2^ = 0.17; BF_10_ = 0.12). These results confirm the absence of sex differences in the reward liking between pre- and post-experiment (notice: the BF_10_ = 1.4 indicates anecdotal evidence^32–34^, which is not supported by all the other statistic values).

The results of the ANOVA on the wanting scores showed a significant effect of time and strong Bayesian evidence in favor of the alternative hypothesis (*F*_1,39_ = 31.39; *p* < 0.001; *η*_p_^2^ = 0.45; BF_10_ = 8.72 × 10^4^), consisting in an increase of wanting comparable between outcomes from pre- to post-experiment, which confirms the general increase of hunger of all participants throughout the experiment. The other factors were not significant (sex: *F*_1,39_ = 0.97; *p* = 0.33; *η*_p_^2^ = 0.02; BF_10_ = 0.53; time by outcome interaction: *F*_2,78_ = 0.27; *p* = 0.76; *η*_p_^2^ = 0.01; BF_10_ = 0.08; time by sex interaction: *F*_1,39_ = 0.01; *p* = 0.94; *η*_p_^2^ < 0.001; BF_10_ = 0.16; outcome by sex interaction: *F*_2,78_ = 0.46; *p* = 0.63; *η*_p_^2^ = 0.01; BF_10_ = 0.12; time by outcome by sex interaction: *F*_2,78_ = 0.87; *p* = 0.42; *η*_p_^2^ = 0.02; BF_10_ = 0.17). In summary, these results confirm absence of difference between the two groups in liking and wanting.

#### Explicit measures of Pavlovian and instrumental learning phases

During the first Pavlovian learning phase, all participants (both males and females) successfully achieved the learning criterion. Specifically, all participants answered correctly and did not require any further repetition beyond the minimum two required.

During the instrumental learning phase, all participants successfully achieved the learning criterion. Forty participants (95.2%) always answered correctly and did not require any further repetition beyond the minimum two required, 2 participants (4.8%) got questions wrong once and had to repeat the blocks three times.

During the second Pavlovian learning phase, all participants successfully achieved the learning criterion. Forty participants (95.2%) always answered correctly and did not require any further repetition beyond the minimum two required. One participant (2.4%) got questions wrong once and had to repeat the blocks three times. One participant (2.4%) got questions wrong twice and had to repeat the blocks four times.

#### Instrumental learning phase

**Fig. 1:**
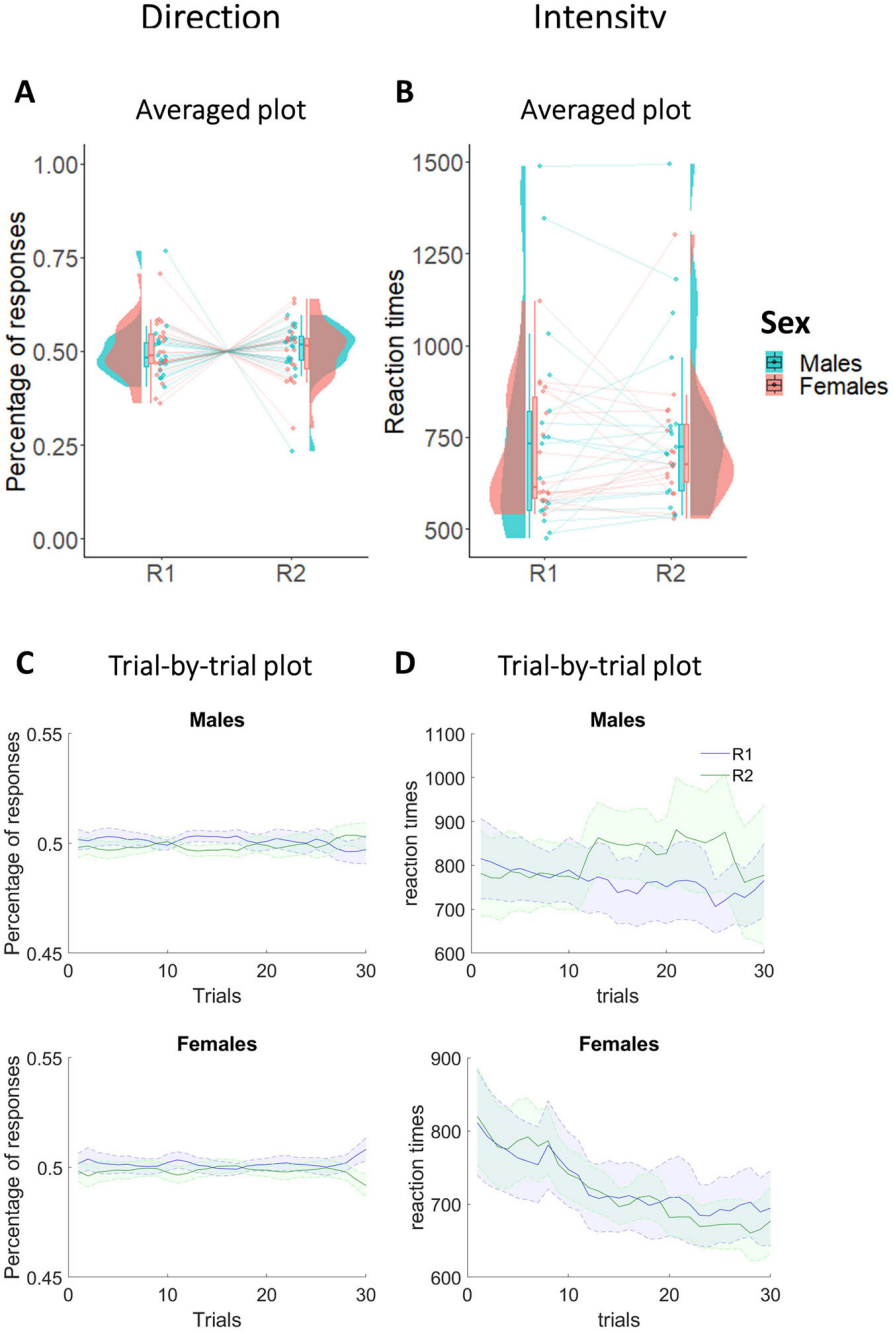
Percentage of responses and reaction times across males and females in the Instrumental learning phase. Direction plots respectively show averaged (**A**) and trial-by-trial (**C**) percentage of R1 and R2 in males and females. Intensity plots respectively show averaged (**B**) and trial-by-trial (**D**) reaction times of R1 and R2 in males and females. In both averaged graphs, boxplots, individual scores, and data distributions are reported in coral for females and blue for males. In trial-by-trial graphs, *x*-axis always represents trials. *Y*-axis represents the percentage of (**C**) and the reaction times (**D**) of R1 (blue) and R2 (green). The shaded regions reflect ±SEM (standard error of the mean) across subjects for each response. Data are smoothed using a 15-trial moving average. Overall, data show absence of differences between R1 and R2 and between males and females.

To exclude sex differences in the total number of responses performed, as well as initial preferences for R_1_ and R_2_ in the instrumental learning phase, we performed two 2 × 2 mixed-measures ANOVAs with response (R_1_/R_2_) as within-subjects factor, and sex (Males/Females) as between-subjects factor, respectively using the percentage of responses (Fig. 1A, C) and reaction times (Fig. 1B, D) as dependent variables. Due to technical problems, reaction times were not recorded in the first 7 participants, so reaction times refer to a sample of 35 participants. For the percentage of responses, results showed Bayes factors in favor of the null hypothesis and no statistically significant main effects of response (*F*_1,40_ < 0.001; *p* = 0.98; *η*_p_^2^ < 0.001; BF_10_ = 0.23), sex (*F*_1,40_ < 0.001; *p* = 0.99; *η*_p_^2^ < 0.001; BF_10_ = 0.29), or interaction between response and sex (*F*_1,40_ < 0.001; *p* = 0.91; *η*_p_^2^ < 0.001; BF_10_ = 0.25). These results are confirmed even for the reaction times, both for the main effects (response: *F*_1,33_ = 0.35; *p* = 0.56; *η*_p_^2^ = 0.01; BF_10_ = 0.29; sex: *F*_1,33_ = 0.77; *p* = 0.39; *η*_p_^2^ = 0.02; BF_10_ = 0.56) and for the interaction between response and sex (*F*_1,33_ = 0.07; *p* = 0.79; *η*_p_^2^ = 0.002; BF_10_ = 0.33). In summary, these results confirm absence of difference between the two groups in Instrumental learning.

#### Transfer phase

**Fig. 2:**
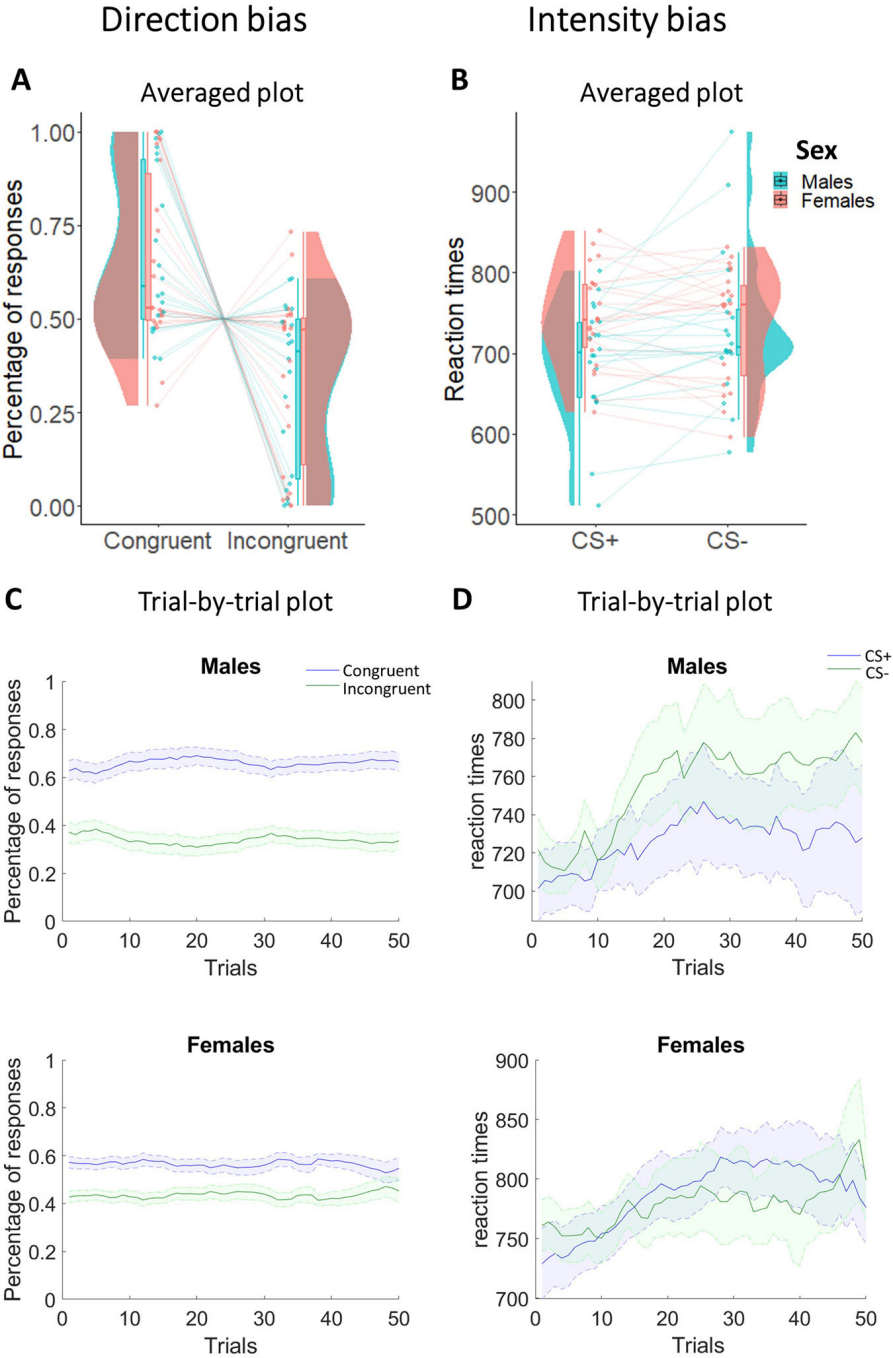
Percentage of responses and reaction times across males and females in the transfer phase. Direction bias plots respectively show averaged (**A**) and trial-by-trial (**C**) percentage of congruent and incongruent responses in males and females. Intensity bias plots respectively show averaged (**B**) and trial-by-trial (**D**) reaction times to CS+ and CS− in males and females. In both averaged graphs, boxplots, individual scores, and data distributions are reported in coral for females and blue for males. In trial-by-trial graphs, *x*-axis always represents trials. *Y*-axis represents the percentage of congruent (blue) and incongruent (green) responses for the direction bias, and the reaction times to CS+ (blue) and CS− (green) for the intensity bias. The shaded regions reflect ±SEM (standard error of the mean) across subjects for each response. Data are smoothed using a 15-trial moving average. Overall, data show the direction bias (greater percentage of congruent responses) in both sexes, while the intensity bias (faster reaction times to CS+ than CS−) is present in males, and not in females.

**Fig. 3:**
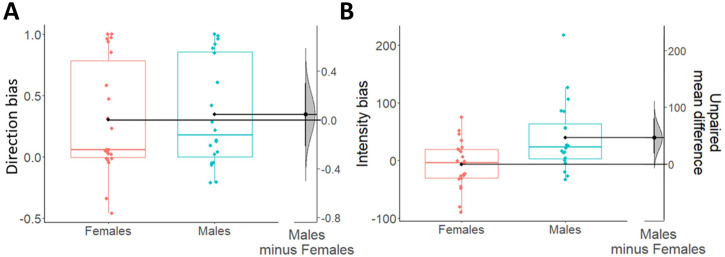
Direction and intensity biases during transfer phase (appetitive context). Boxplots show direction (**A**) and intensity (**B**) biases indexes, respectively scaled on their own left *y*-axes. Females are shown in coral and males are shown in blue. For all boxplots, the centre line represents the median, the box represents the interquartile range (IQR), whiskers indicate 1.5∗IQR and the plotted points represent participants. Single black dots (one for each box) represent the means of each group of data. On the right side of each figure (**A**, **B**), the unpaired mean differences (derived from a bootstrap sampling distribution of 5000 samples) between males and females are plotted and scaled on their right *y*-axes. 95% confidence intervals are indicated by black vertical error bars. Overall, the data show no sex differences in the direction bias, and an increased intensity bias in males as compared to females. Analyses performed excluding the male outlier in the intensity bias (blue point out of whiskers) gave the same results as those shown there.

For the direction bias, we performed a 2 × 2 mixed-measures ANOVA with response (Congruent/Incongruent) as within-subjects factor, sex (Males/Females) as between-subjects factor, and the percentage of responses as dependent variable (Fig. 2A, C). Results showed a statistically significant main effect response and strong Bayesian evidence in favor of the alternative hypothesis (*F*_1,40_ = 21.39; *p* < 0.001; *η*_p_^2^ = 0.35; BF_10_ = 2.34 × 10^6^). Specifically, participants of both sexes showed a greater number of congruent responses (males: *M* = 0.67; SD = 0.22; females: *M* = 0.65; SD = 0.23) compared to incongruent responses (males: *M* = 0.33; SD = 0.22; females: *M* = 0.35; SD = 0.23). Estimation statistics (Fig. 3A) confirmed the absence of differences between males and females in choice direction (Δ_mean_ = 0.05, 95% CI [−0.22 0.3]). All other factors were not statistically significant and presented Bayesian evidence in favor of the null hypothesis (sex: *F*_1,40_ < 0.001; *p* = 0.99; *η*_p_^2^ < 0.001; BF_10_ = 0.25, response by sex interaction: *F*_1,40_ = 0.11; *p* = 0.75; *η*_p_^2^ = 0.003; BF_10_ = 0.31).

For the intensity bias, we used a 2 × 2 mixed-measures ANOVA with CS (CS+/CS−) as within-subjects factor, sex (Males/Females) as between-subjects factor, and reaction times as dependent variable (Fig. 2B, D). Results showed a significant main effect of CS not supported by Bayesian evidence (*F*_1,40_ = 4.14; *p* = 0.049; *η*_p_^2^ = 0.09; BF_10_ = 0.8), a statistically significant interaction between CS and sex supported by moderate Bayesian evidence in favor of the alternative hypothesis (*F*_1,40_ = 8.61; *p* = 0.01; *η*_p_^2^ = 0.18; BF_10_ = 7.46), and no statistically significant main effect of sex with Bayesian evidence in favor of the null hypothesis (*F*_1,40_ = 1.72; *p* = 0.2; *η*_p_^2^ = 0.04; BF_10_ = 0.75). More specifically, only males showed faster reaction times to the CS+ (*M* = 692.13; SD = 72.1) compared with the CS− (*M* = 731.47; SD = 92.63), while females did not report differences in reaction times between CS+ (*M* = 743.57; SD = 60.95) and CS− (*M* = 736.45; SD = 69.4). This result was confirmed by estimation statistics showing the presence of an intensity bias in males but not in females. In line with this, Fig. 3B shows an average intensity bias index around 0 in females only (indicating the absence of effect in this group), and a positive difference in intensity bias between males and females (Δ_mean_ = 46.5, 95% CI [18.7 80.5]).

#### Interim discussion

Overall, the results of experiment 1 showed that appetitive cues bias the direction of instrumental actions similarly in males and females. In contrast, a difference emerged in the intensity bias on instrumental actions. Specifically, only males presented invigoration of actions while appetitive stimuli were presented. On the contrary, females’ action intensity did not seem to be affected by the presence of an appetitive cue, so the intensity of their actions (i.e., reaction times) was independent of the motivational value of the cue.

However, a crucial open issue remains. The results of our experiment do not clarify whether the observed sex differences depend on a general arousal state (a “central motivational state”, as defined by Rescorla and Solomon^35^) or on the valence (positive or negative) of the cue. It is well known that an intensity bias can be generated by a general increase in motivation triggered by the cue, which can be seen in both appetitive and aversive contexts even if with opposite consequences (the invigoration of instrumental actions can allow both to approach rewards or avoid punishments)^2^. Nevertheless, PIT studies^36,37^ showed that the neural areas and the mechanisms involved in aversive contexts only partially overlap with those observed in appetitive contexts. Moreover, past studies on sex differences have shown an increased sensitivity to aversive outcomes in females^38,39^, which might be reflected in aversive cues biases. Therefore, to check whether our results could be extended to aversive biases, we re-analyzed data from a previously published aversive Pavlovian-to-Instrumental Transfer study^15^ adding sex as an independent variable.

### Experiment 2

#### Pavlovian learning phase

**Fig. 4:**
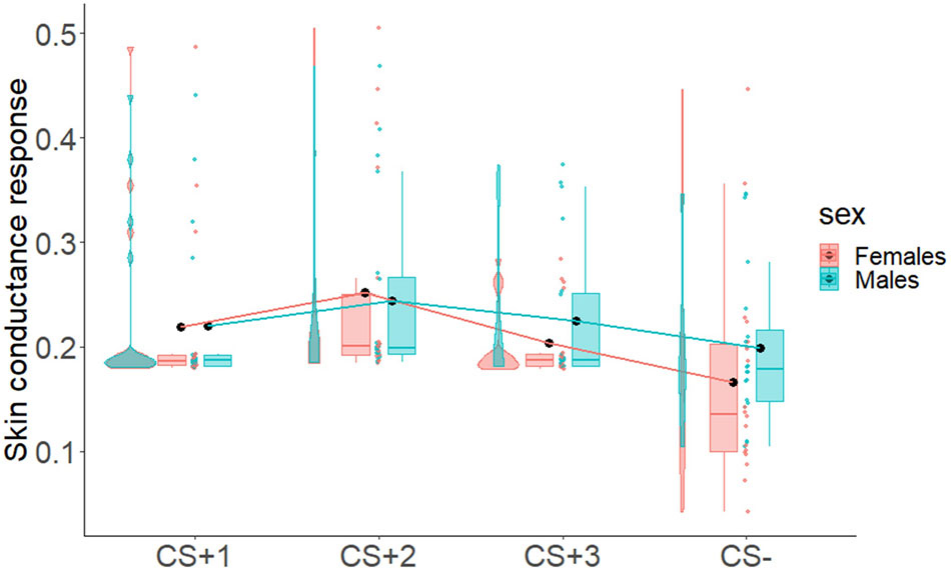
Acquisition of Pavlovian learning in males and females. The figure shows the skin conductance response (SCR) to the CSs (CS+_1_, CS+_2_, CS+_3_, CS−) in males and females during the Pavlovian learning phase. Boxplots, individual scores, and data distributions are reported in coral for females and blue for males. Black dots represent means. Overall, data show an increased SCR to the three CSs+, as compared to the CS−, in both males and females.

To assess successful implicit acquisition of Pavlovian learning in both sexes, we performed two 4 × 2 mixed-measures ANOVAs with CS (CS+_1_, CS+_2_, CS+_3_, CS−) as within-subjects factor, and sex (Males/Females) as between-subjects factor, using the skin conductance response (SCR) as dependent variable. For this analysis, we considered only the second hemiblock of this phase for each CS, i.e., trials in which participants have learned the value of CSs+ and CS− and should have shown differences in SCR. Results showed a statistically significant main effect of CS and strong Bayesian evidence in favor of the alternative hypothesis (*F*_3,108_ = 6.25; *p* < 0.001; *η*_p_^2^ = 0.15; BF_10_ = 42.49). All other factors were not statistically significant and presented Bayesian evidence in favor of the null hypothesis (sex: *F*_1,36_ = 0.42; *p* = 0.52; *η*_p_^2^ = 0.01; BF_10_ = 0.31, CS by sex interaction: *F*_3,108_ = 0.75; *p* = 0.53; *η*_p_^2^ = 0.02; BF_10_ = 0.15). Figure 4 shows an increase of SCR in both males and females for the three CS+, as compared to CS−.

#### Transfer phase

**Fig. 5:**
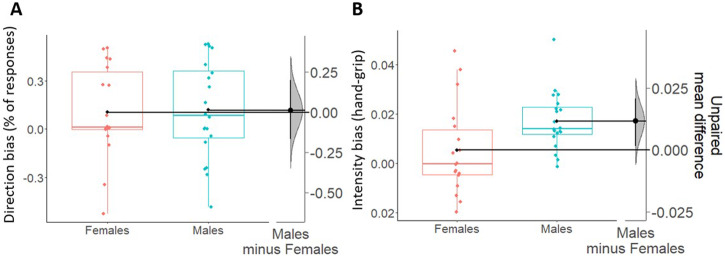
Direction and intensity biases during transfer phase (aversive context). Boxplots show direction (**A**) and intensity (**B**) biases, respectively scaled on their own left *y*-axes. Females are shown in coral and males are shown in blue. For all boxplots, the centre line represents the median, the box represents the interquartile range (IQR), whiskers represent 1.5∗IQR and the plotted points represent participants. Single black dots (one for each box) represent the means of each group of data. On the right side of each figure (**A**, **B**), the unpaired mean differences (derived from a bootstrap sampling distribution of 5000 samples) between males and females are plotted and scaled on their right *y*-axes. 95% confidence intervals are indicated by black vertical error bars. Overall, data show no sex differences in the direction bias, and an increased intensity bias in males as compared to females. Analyses performed excluding the outliers in the intensity bias (one female and one male out of whiskers) gave the same results as those shown there.

For the direction bias (Fig. 5A), no differences (*t*_36_ = −0.12; *p* = 0.9; *d*_cohen_ = −0.04; BF_10_ = 0.32) were found between males (*M* = 0.12; SD = 0.3) and females (*M* = 0.11; SD = 0.29). Moreover, estimation statistics clearly show that the mean difference between the two groups in direction bias is centered around 0 (Δ_mean_ = 0.01, 95% CI [−0.17 0.2]).

For the intensity bias (Fig. 5B), a significant increase and weak Bayesian evidence in favor of the alternative hypothesis (*t*_36_ = −2.39; *p* = 0.02; *d*_cohen_ = −0.78; BF_10_ = 2.73; Δ_mean_ = 0.012, 95% CI [0.001 0.02]) was found in males (*M* = 0.02; SD = 0.01) as compared to females (*M* = 0.005; SD = 0.02). In line with this, Fig. 5B clearly shows that the grip force in females overlaps with 0 (thus indicating absence of effect) while it is above 0 in males.

## Discussion

A broad range of interindividual differences characterize motivated behaviors, both in humans and non-human animals^10,40^. The purpose of the present study was to test sex differences in how motivational outcome-predictive cues bias the direction and intensity of instrumental actions. To this end, we compared the direction and intensity biases exhibited by males and females in two Pavlovian-to-Instrumental Transfer paradigms, respectively conducted in an appetitive (experiment 1) and an aversive context (experiment 2).

In both experiments, results showed that sex can modulate the intensity of instrumental actions. Specifically, the intensity of the instrumental actions (faster reaction times, stronger grip force) was enhanced by motivational cues (as compared to neutral cues) in males only. Conversely, females did not present such bias, so their instrumental actions were performed with the same intensity in the presence of motivational and neutral cues. The direction bias was instead not modulated by sex, i.e., both males and females favored actions congruent with the cue-predicted outcome, compared to incongruent actions.

Critically, such male-selective intensity bias emerges despite both males and females having explicitly learned the stimulus-outcome (Pavlovian learning) and response-outcome (instrumental learning) associations to a comparable degree. Moreover, the difference in intensity bias cannot be attributed to either higher vigor or higher outcome reactivity in males compared to females^26,38^, as during both instrumental learning and transfer phases comparable overall (i.e., regardless of response and cue, respectively) reaction times were reported. Crucially, sex differences emerged only during the transfer phase and in the presence of a motivational cue, compared to a neutral one, even if not followed by an outcome (under extinction condition). This suggests that our result can be selectively attributed to the incentive properties that motivational cues exerted on the intensity of instrumental actions^6^.

The convergence of results between experiment 1 and experiment 2 further demonstrates that the observed sex differences are valence-independent, with males reporting a stronger intensity bias in both appetitive and aversive contexts. The intensity bias can thus manifest itself in terms of the invigoration of actions directed to obtain rewards (in appetitive contexts) and avoid punishments (in aversive contexts)^2,6^. This result provides further evidence to the incentive salience interpretation of the intensity bias^35,41,42^. Moreover, the successful implicit acquisition (skin conductance response) of Pavlovian learning for both sexes in Experiment 2 suggests that the absence of the intensity bias in females is not due to a lack of acquisition of incentive salience by the cue but rather to a lack of use of such information to guide choice, at least in aversive contexts. Unfortunately, the absence of psychophysiological measures in Experiment 1 does not allow to extend this interpretation to the appetitive context. Despite the pitfalls of using psychophysiological indexes in appetitive Pavlovian learning^43^, future studies should try to address this open question.

Overall, these results are in line with previous research conducted in humans which found that males are characterized by greater subjective arousal ratings, behavioral accuracy, skin conductance response, and higher activity in the nucleus accumbens and midbrain regions in response to high-salience cues, independent of their valence^27^. Moving from this evidence, we can hypothesize that the sex differences observed in the present study may reflect behavioral and neural differences in the mechanisms underlying the processing of motivational stimuli in males and females. For example, previous studies hypothesized the role played by dopaminergic circuits in the intensity bias^44,45^, with a crucial involvement of subcortical areas like the central nucleus of the amygdala^8^ and the nucleus accumbens core^46^. However, future studies will have to address this question more directly.

Concerning the direction bias, we did not find any modulation of sex, i.e., both males and females favored actions congruent with the outcome predicted by the cue, as compared to incongruent actions. Despite sharing an important motivational basis with the intensity bias^1,46^, the direction bias has been reported to require high-level cognitive abilities, such as working memory^10^ and conscious perception of the cues^13,14^, as well as the activation of cortical structures as the lateral prefrontal cortex^11^. The presence of this bias in both sexes may suggest that people, regardless of sex, exploit the informative value of external cues to guide the direction of their decisions^11^. However, the absence of differences among sexes does not exclude possible differences in the strategies and the neural circuits that mediate the direction bias in males and females^22,24,25^. In this regard, for example, Barker and colleagues^29^ reported no sex differences in terms of motivational bias on instrumental actions in mice; however, a positive correlation between such bias and ethanol-seeking was found in males only, thus supporting the idea that different underlying processes could be involved in males and females. Lastly, we cannot rule out the possibility that our task is too simple to detect sex differences (i.e., ceiling effect) in the direction bias, which could be revealed by more complex tasks, for example involving a higher number of responses, cues, or outcomes.

Finally, the evidence presented in this study has important clinical implications. How humans interpret and react to affect-laden environmental cues may influence the development of substance use disorder or mental illness. Although our task does not allow distinguishing between adaptive and maladaptive forms of cue-guided decisions, the direction and intensity biases have been widely linked to maladaptive behaviors involving alteration of dopaminergic circuits^47^, with a particular involvement of amygdala^48^—like addiction^49,50^—which are typically characterized by a greater prevalence^51,52^ and vulnerability^29,53^ in males. Further studies are needed to clarify whether and how the higher intensity bias in males plays a role in the acquisition, maintenance, and relapse of addiction, thus contributing to sex discrepancies in prevalence and vulnerability. Similarly, further research is required to investigate the possible role of male-selective intensity bias in pathologies involving alterations of motivated behavior, such as depression and anxiety disorders, for which sex differences have often been observed^54–56^.

In conclusion, this investigation revealed sex differences in how motivational biases affect instrumental responding in humans. Our study demonstrates that a crucial part of our everyday behavior, i.e., the intensity of instrumental actions aimed at obtaining reward or avoiding punishments, can be particularly affected in males presented with outcome-predictive cues during choice. These results set a framework for investigating sex differences in the motivational influences of decision-making, and their adaptive and maladaptive implications. Moreover, such findings can provide suggestions for more personalized treatments of mental disorders characterized by maladaptive motivational decision-making, which should consider sex as a key variable for the processes involved in the development, maintenance, and relapse of such conducts.

## Methods

### Experiment 1

#### Participants

Twenty-two women (mean age = 23.41; sd = 2.68 years) and 20 men (mean age = 23.70; sd = 2.34 years) with no history of neurological or psychiatric diseases voluntarily took part in the experiment. The recruitment process involved posting study announcements on relevant social networks groups and platforms. Interested individuals who responded to these posts were contacted for further screening and potential inclusion in the study. Specifically, potential participants were asked to report eventual history of neurological or psychiatric diseases, and if so, they were excluded from recruitment. All participants gave their written informed consent to take part in the experiment. The study was conducted in accordance with institutional guidelines and the 1964 Declaration of Helsinki and was approved by the Bioethics Committee of the University of Bologna (Prot. 201070, 24/09/2020). The number of participants was established based on a power analysis conducted on MorePower 6.0^57^, for the planned 2 × 2 mixed-measures ANOVA, i.e., the design for both intensity bias and direction bias analyses. Specifically, the following parameters were used: Repeated Measures design factors = 1 factor (CS for intensity bias; Response for direction bias) with 2 levels (CS+, CS− for intensity bias; congruent, incongruent for direction bias); Independent Measures design factors = 1 factor (Sex) with 2 levels (Males, Females); Dependent Measures effect of interest = 1 factor with two levels; Independent Measures effect of interest = 1 factor with 2 levels; effect size (*η*_p_^2^) = 0.17; significance level = 0.05; power = 0.8. The effect size was estimated based on the mean of effect sizes (*η*_p_^2^) obtained by previous studies conducted with a similar task^10,11,13,15,40^. In this study, we refer to “sex differences” because we categorized participants based on their sex, intended as a biological composition at birth, and not based on their gender identity. Specifically, participants were explicitly asked about their assigned sex at birth, rather than their gender identity.

#### Stimuli and procedure

The appetitive Pavlovian-to-Instrumental transfer (PIT) paradigm was divided into four phases: (1) Pavlovian learning phase, in which stimulus-outcome associations were learned; (2) Instrumental learning phase, in which response-outcome associations were learned; (3) Pavlovian learning phase, in which the same stimulus-outcome associations were presented again and strengthened; (4) Transfer phase, in which the influence of conditioned stimuli (cues) on direction and intensity of instrumental responses was tested.

In all task phases, the image of a slot machine was presented in the middle of a computer screen on a white background. The slot machine presented two black displays (one on the top and one on the bottom) and two buttons. On the upper display, when necessary, one of four different colors (red, blue, yellow, green) appeared, corresponding to the four conditioned stimuli (CSs). Two CSs (CS+_1_ and CS+_2_) were necessary to study the direction bias, and the other two CSs (CS+_3_ and CS−) allowed to study the intensity bias. On the lower display, three food snacks used as separate rewarding outcomes were presented, as well as the non-rewarding outcome (white “X”). The three rewards were tailored to each participant: upon recruitment, participants rated the subjective liking of a set of 21 different food items (10 savory foods: chips, flatbreads, savory biscuits, almonds, peanuts, breadsticks, crackers, twisties, nachos, savory taralli; 11 sweet foods: mars, orociock, gaufrettes, small plum cakes, croissants, jelly beans, Nutella, tarts, chocolate bars, sweet biscuits, protein bars). For each participant, the experimenter selected three foods that were associated with comparable (±1) liking score on a 5-point Likert scale ranging from 0 (Not at all) to 5 (Very much). Images corresponding to these foods were later used as indicative of the type of rewards during the task. The task was programmed using OpenSesame (version 3.2)^58^.

Participants were asked to refrain from eating for 3 h prior to the experiment. To ensure comparable values between the three rewards at the time of the experiment, a new liking and wanting 9-point Likert scale was presented right before the beginning of the task. If the participant expressed a preference for one reward over the others, such reward would be substituted with a comparable one (percentage of choice of savory/sweet snacks: Males = 40/60; Females = 53/47). The three selected rewards were placed within sight, near the computer screen, and remained visible throughout the entire duration of the experiment to ensure a high level of motivation. Participants were then asked to rate their current level of hunger, in order to exclude initial group differences in such variable. The statistical analysis (independent sample *t*-test, *t*_40_ = 0.47; *p* = 0.64; *d*_cohen_ = 0.15; BF_10_ = 0.33) showed no differences in hunger between the two groups.

The experimental session lasted about 1 h and 45 min and participants could rest between the phases to prevent fatigue and loss of attention. Participants were informed that, at the end of the experiment, they would receive an amount of food proportional to the number of food pictures visualized during all task phases. Then, the experimental session would begin. In each phase, participants were required to pay attention to the screen and follow the instructions reported at the beginning of each task phase. At the end of the experiment, participants were provided with at least one of each previously selected reward (i.e., at least three distinct rewards).

#### PIT task

##### Pavlovian learning phase

**Fig. 6:**
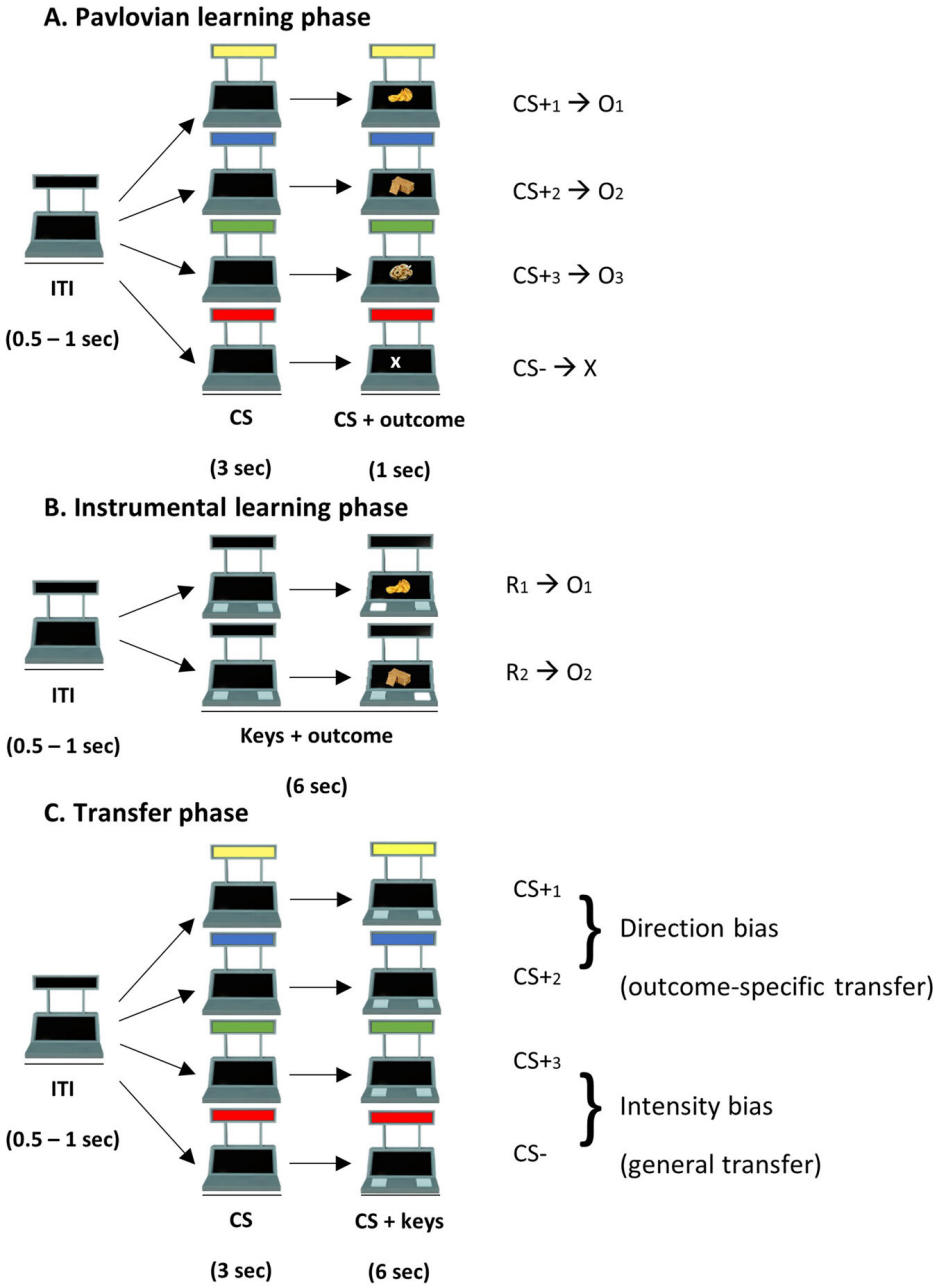
PIT task. **A** Pavlovian learning phase. In each trial, after an intertrial interval (ITI) of 0.5–1 s, one of four cues (CSs) was presented, followed by one of four different outcomes. Three CSs were paired with three different rewarding outcomes, while the fourth control CS (CS−) was paired with a no-rewarding outcome. Participants had to learn the cue-outcome associations. **B** Instrumental learning phase. In each trial, after an ITI of 0.5–1 s, two buttons appeared on the slot machine. Participants were free to press one of the two buttons for 6 s. After each press, the outcome was presented on the lower screen of the slot machine for 1 s. the two responses (R1, R2) were associated with two different rewarding outcomes. Participants had to learn the response-outcome associations. **C** Transfer phase. In this phase, the influence of the CSs on the instrumental responses was tested. In each trial, after an ITI of 0.5–1 s, one of the CSs was presented for 3 s. Then, the two buttons appeared and participants were free to press one of the two buttons for 6 s. CS+_1_ and CS+_2_ allowed testing the direction bias, and CS+_3_ and CS− allowed testing the intensity bias.

**Fig. 7:**
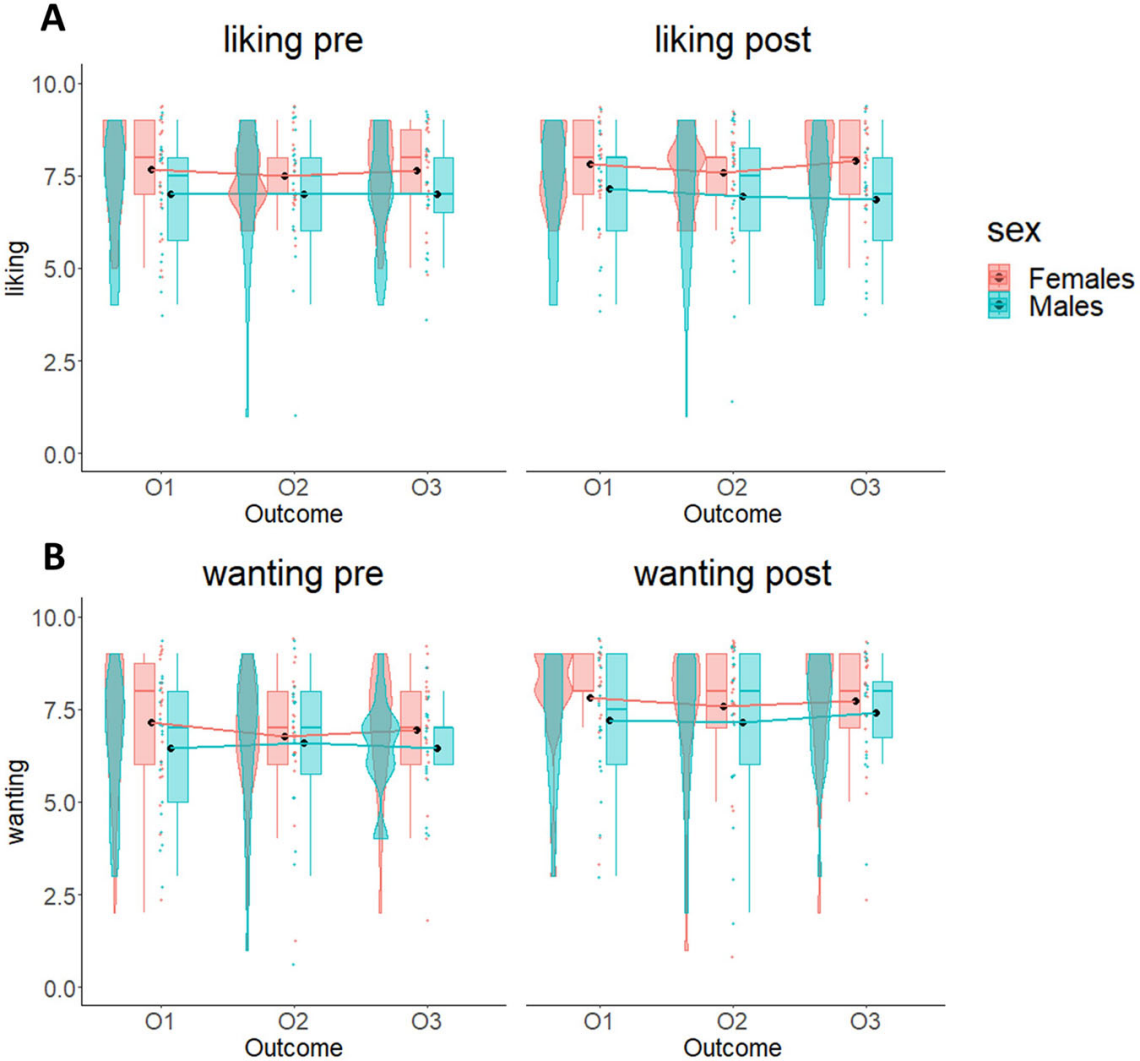
Liking and wanting scores across males and females. The two plots show liking (**A**) and wanting (**B**) scores for the three selected outcomes (O_1_, O_2_, O_3_) before and after Pavlovian learning phase. In both graphs, boxplots, individual scores, and data distributions are reported in coral for females and blue for males. Black dots represent means. Overall, data show absence of differences between O_1_, O_2_ and O_3_, and between males and females.

In this phase (Fig. 6A), participants were required to learn the association between four conditioned stimuli and their respective outcomes. Three of them (CS+_1_, CS+_2_, CS+_3_) were paired with the three individually-tailored rewarding outcomes previously selected (O_1_, O_2_, O_3_). These CSs we associated with their respective reward in 80% of trials and with a non-rewarding outcome (“X”) in the remaining trials. The fourth stimulus (CS−) was always paired with the non-rewarding outcome (“X”). Each trial consisted of a variable inter-trial interval (ITI, 0.5–1 s), in which the slot machine was “empty” (with no colors or rewards), followed by the appearance of one of the CSs (3 s). After this period, the corresponding outcome appeared simultaneously to the CS for 1 additional second. During this phase, no responses were available. Trials proceeded in pseudo-random order, such that no more than five consecutive CSs of the same type occurred in a row, in order to exclude the potential effect of unbalanced trial order among different stimuli (Fig. 7).

Learning criterion: the task was structured in a series of blocks that allowed for the Pavlovian learning phase to proceed until a specific learning criterion was reached. The first block consisted of 180 trials (45 for each CS). At the end of the block, the question “What food did you win with this color?” appeared (one for each CS) to test whether all stimulus-outcome associations were correctly established. After an additional block of 20 trials (5 trials for each CS), the questions appeared again and, if the participant answered correctly both times, the learning criterion was achieved, otherwise 20 additional trials (5 for each CS) were presented again up to a maximum of four times. Hence, the learning criterion consisted of correctly reporting the stimulus-outcome associations at least two times in a row. When the learning criterion was achieved, the task moved to the following phase. After four wrong answers, the task was aborted.

##### Instrumental learning phase

In this phase (Fig. 6B), participants were required to learn the association between two possible responses (R_1_, R_2_) and their respective rewarding outcomes (O_1_, O_2_). These outcomes were the same as those associated with two of the four stimuli presented during the Pavlovian learning phase (CS+_1_ and CS+_2_). The response consisted of pressing one of two possible computer keys, corresponding to the two buttons appearing on the slot machine. Each time a computer key was selected, the corresponding button on the slot machine appeared illuminated and pressed. After each response, the corresponding outcome appeared for 1 s, during which no response was possible. All trials lasted 6 s. On each trial, participants were instructed to press only one of the two buttons as many times as they wished. Importantly, participants had the opportunity to win multiple rewarding outcomes within a single trial, as responses were reinforced following a variable ratio reinforcement schedule consisting in a 25% chance of receiving the reward and 75% chance of getting the non-rewarding outcome (“X”). During the intertrial interval (ITI) the slot machine was still visible, but the buttons disappeared so that the response options were not available for a jittered duration ranging between 0.5 and 1 s. Before starting the task, six practice trials with non-rewarding outcomes were presented to familiarize the participant with the link between the computer key and the corresponding slot machine button.

Learning criterion: the task was structured in a series of blocks that allowed for the instrumental learning phase to proceed until a specific learning criterion was reached. Each block terminated after a total of 30 rewards were obtained, to ensure at least 15 repetitions of the response-outcome association for each reward type. At the end of each block, the question “What food did you win by pressing this button?” appeared (one for each response) to test whether all response-outcome associations were correctly established. These blocks were repeated from a minimum of two times to a maximum of eight times. The learning criterion consisted of correctly reporting the response-outcome associations at least two times in a row. If the learning criterion was achieved, the task moved to the following phase. After four wrong answers, the task was aborted.

##### Second Pavlovian learning phase

This phase was structured exactly as the first Pavlovian learning phase. It served to recall and strengthen the previously acquired stimulus-outcome associations.

##### Transfer phase

This phase (Fig. 6C) aimed to test the influence of the CSs on instrumental responding. Each trial was structured as follows: first, an empty slot machine (with no CSs, buttons, or rewards) appeared for a variable ITI length (0.5–1 s); then, one of the task-irrelevant CSs (CS+_1_, CS+_2_, CS+_3_, or CS−) appeared on the slot-machine for 3 s. Finally, the two buttons previously trained during instrumental learning appeared along with the CS for 6 s. On each trial, participants were instructed to press only one of the buttons as many times as they wished. Very rarely did participants press one key in error and then switched to the other during the response time (number of errors for men: *M* = 0.55; SD = 0.89; women: *M* = 0.45; SD = 0.69). We retained these data for our analyses, as we assumed that these were probably unintentional errors. Only one female participant had a significantly higher number of errors (*N* = 54). We repeated our main analyses without this participant and the pattern of results did not change.

This phase consisted of a total of 200 trials (50 for each CS). Trials proceeded in pseudo-random order, such that no more than five consecutive CSs of the same type occurred in a row, in order to exclude the potential effect of unbalanced trial order among different stimuli.

The whole transfer phase was conducted under extinction, so no rewards were shown. This allowed us to test the influence of CSs on instrumental responding without the confounding effect of the reward. Specifically, we employed a “nominal extinction” procedure in which participants were instructed that they were still winning but, since the lower display of the slot machine was malfunctioning, they would not be able to see the outcomes^12,59,60^.

The rationale of this phase was to test the direction (also known as, outcome-specific transfer) and the intensity (also known as general transfer) biases respectively using as dependent variables the percentage of responses and reaction times^6,15,16^. Two different trial types were designed to disentangle the two biases.

Specifically, in the direction bias trials, participants were required to choose between R_1_ or R_2_ while CS+_1_ or CS+_2_ were presented. R_1_ and R_2_ were previously paired with two different rewarding outcomes, of which only one was also previously paired with the concurrently presented CS. So, if the CS+_1_ was presented, choosing R_1_ would constitute a congruent response, while choosing R_2_ would constitute an incongruent response. Similarly, if the CS+_2_ was presented, choosing R_1_ would constitute an incongruent response, while choosing R_2_ would constitute a congruent response. Evidence for the direction bias would be seen if the presence of the CS induced a higher number of congruent responses, as compared to incongruent responses.

In the intensity bias trials, participants were required to choose between R_1_ or R_2_ while CS+_3_ or CS− were presented. Importantly, none of the responses was compatible with the CS currently available (i.e., during the previous phases, these CSs were respectively associated with a different or no outcome). Evidence for the intensity bias would be seen if participants had faster reaction times during the presentation of a CS+_3_ (thereafter we will refer to CS+_3_ as “CS+”), as compared to the CS−.

Typically, the intensity bias is conceptualized as an invigoration of responses in the presence of CS+, also detectable through higher percentage of responses (both R_1_ and R_2_), as compared to CS−. Nevertheless, recent studies that used the percentage (or number) of responses as dependent variable failed to find the intensity bias^61^ or obtained unclear results^16^. Similarly, the direction bias was often not detected by using reaction times as dependent variable^62,63^. In line with these results, a double dissociation between the direction and the intensity bias has been found, with the first one detectable through number of responses and not through hand-grip, and vice versa the second (intensity bias) detectable through hand-grip and not through number of responses. Following these results, a growing body of research started to use separated measures for these two biases^64–66^, operationalizing the intensity bias by using more direct measures of the vigor exerted for each response. Based on these studies, we used reaction times to test intensity bias. However, we also performed the analyses on the percentage of responses and on the response rate for the intensity bias, as well as the analyses on reaction times for the direction bias (see Supplementary Materials).

#### Data analysis

The direction bias was tested by comparing the percentage of congruent (i.e., consistent with the outcome predicted by the cue) and incongruent (i.e., not consistent with the outcome predicted by the cue) responses in both males and females. The intensity bias was tested by comparing the vigor (reaction times) of the responses performed while presented with a reward-associated conditioned stimulus (CS+) and with a neutral stimulus associated with no reward (CS−).

Data were processed offline using custom-made MATLAB scripts (The MathWorks, Inc., Natick, MA, USA) and statistical analyses were performed with RStudio v1.0.136 (RStudio Team, 2016). For the analysis of the intensity bias, we considered the reaction time of the first response in each trial, and excluded trials in which the first response was given beyond two seconds after the appearance of the buttons (number of trials excluded for CS: *M* = 1.54; SD = 2.12). We then calculated individual mean and standard deviations and excluded outlier trials beyond two standard deviations from the mean for each participant (number of trials excluded for CS: *M* = 2.99; SD = 0.89)^67^. Normality and sphericity assumption of the data were visually inspected and verified through low values of skewness and kurtosis for all variables^68^. Finally, we used the r boxplot function to detect outliers, defined as the values outside 1.5*interquartile range, which revealed one outlier value. We performed the analysis including the outlier (although we obtained the exact same pattern of results with and without this outlier).

In line with the state-of-the-art recommendation for good methodological and statistical practices^69,70^, the statistical analyses were conducted following a model multiverse approach analysis, in order to provide converging evidence for the reported effects. Specifically, a Fisherian null hypothesis significance testing (NHST) approach was used to establish the presence of statistically significant (*p* < 0.05) differences between the considered conditions. Effect sizes were estimated with Cohen’s *d* for all *t*-tests and partial eta squared for all ANOVAs. In addition, a Bayesian approach was used to determine the weight of the experimental hypothesis with respect to the null hypothesis. In particular, the Bayes Factor (BF_10_) is reported as the probability associated with the alternative hypothesis (H_1_) over the null hypothesis (H_0_)^71^. A flat (or non-informative) prior distribution was used for Bayesian analyses^71^. Following the classification existing in the literature^32^, the BF_10_ can be interpreted as “weak evidence” (1 < BF_10_ ≤ 3), “moderate evidence” (3 < BF_10_ ≤ 10), or “strong evidence” (10 < BF_10_ ≤ 30).

Finally, the difference between the groups was quantified via estimation statistics^72,73^. Specifically, to estimate the difference we used two indices of direction and intensity bias. The direction bias index was computed as the difference between the percentage of congruent and incongruent responses. The intensity bias index was computed as the difference between the vigor (reaction times) of the responses performed while presented with CS− and with CS+. Estimation plots show individual data points for each sex, and the unpaired difference with 95% bias-corrected accelerated confidence interval (CI) based on 5000 bootstrap samples. Unpaired differences across sexes were estimated based on the mean (Δ_mean_). The inference was based on the inspection of the estimated difference across sexes (Δ_mean_) and the precision of such estimate (i.e., length of the CI): intervals including 0 were interpreted as indicative of no evidence of effect; intervals not including 0 were interpreted as indicative of weak, moderate, or strong evidence of effect based on the size of the estimated difference and its precision (the longer the CI, the lower the precision, and the weaker the evidence)^74^. Thus, for both indexes, a higher value corresponded to a greater bias, while 0 corresponded to the absence of the bias.

### Experiment 2

#### Participants

Eighteen women (mean age = 25.33; SD = 6.07 years) and 20 men (mean age = 25.05; SD = 5.50 years) with no history of neurological or psychiatric diseases voluntarily took part in the study. The number of participants was selected for the published study, without considering the hypothesized sex difference. Thus, we conducted an a-priori power analysis adding the sex as a factor. This led to a sample size of 44 participants. The discrepancy between the estimated and actual sample size (*N* = 38) was minimal, and a post-hoc power analysis indicated a power of 0.63.

All participants gave their written informed consent to take part in the experiment. The study was conducted in accordance with institutional guidelines and the 1964 Declaration of Helsinki and was approved by the Department of Psychology Ethics Committee of the University of Cambridge.

#### Stimuli and procedure

The task consisted of an aversive Pavlovian-to-Instrumental Transfer paradigm structured in 3 phases: (1) Instrumental Learning phase; (2) Pavlovian Learning phase; (3) Transfer phase.

In all task phases, the images of five distinct custom-made space scenarios were presented as background on a computer screen. Four of these space scenarios were used as conditioned stimuli (CS+_1_, CS+_2_, CS+_3_, CS−). Two CSs (CS+_1_ and CS+_2_) were necessary to study the direction bias, and the other two CSs (CS+_3_ and CS−) allowed to study the intensity bias. The fifth space scenario was used as background during Instrumental Learning phase. In the central lower part of the screen, a smaller display was employed to exhibit visual feedback. The visual feedback included the phrase “defend yourself” (only during the Instrumental Learning phase), a green circle labeled “missed”, a red triangle labeled “hit”. After the “hit” feedback one of three different aversive noises, each consisting of 100 dB sounds played for 1 s, was consistently presented, serving as unconditioned stimulus (US1, US2 or US3). After the “missed” feedback, no aversive noises were presented. The three noises had been rated as equally aversive and clearly distinguishable by an independent group. In each phase, participants were requested to focus on the screen and follow the instructions. A few example trials were always presented before each phase.

Upon arrival, participants were seated comfortably in a quiet environment, with their position centrally aligned with the screen. A headset, designated for the delivery of aversive sounds during the task, was worn by each participant. Throughout the experiment, data pertaining to galvanic skin response, hand-grip force, and behavioral responses were systematically gathered and subsequently stored for offline analysis.

#### PIT task

##### Instrumental learning phase

Participants were engaged in a space-war game. There were two possible sources of attack, which corresponded to the two aversive noises (or unconditioned stimuli, US1, US2). The two USs were played prior to the beginning of the phase, in order to allow familiarization. For the whole duration of the phase, a single space scenario was presented as background. In each trial, after an ITI of 1.5–2 s, the phrase “defend yourself” appeared to signal the initiation of the trial. Then, for the following 30 s, only one US was randomly presented based on an unpredictable time schedule ranging from 1.5 to 3 s. To avoid attacks, i.e., US1 or US2, participants had to move a joystick toward (respectively) left or right (R1 or R2, counterbalanced between participants), while squeezing a hand-grip. Their task was to figure out and learn the correct response to avoid each specific US. If the US was avoided, the “missed” feedback appeared, and no noises were delivered. This phase lasted about 5 min and included 8 trials (4 for specific US, of about 30 s each).

##### Pavlovian learning phase

Participants were informed that they would now be traveling through different galaxies (corresponding to the four CSs) and that more attacks could be delivered at this stage. In this phase, they were not able to use the joystick to avoid those attacks and were required to learn the association between specific CSs and USs. Two CSs (CS+_1_, CS+_2_) were paired with the same two USs previously used during instrumental learning (US1, US2); a third CS (CS+_3_) was paired with a new US (US3); a fourth CS, serving as CS−, was never paired with any US. All CSs+ were paired with their respective US in the 60% of trials and with no US in the remaining trials.

In each trial, after an ITI of 7–9 s, one of the four CSs (4.5 s) was presented in background, followed by visual feedback (“hit” or “miss”) and one of the USs (or no US, in case of “miss”) for 1 s. This phase lasted about 15 min and included 80 trials (20 for CS).

##### Transfer phase

During this phase, participants had to perform the same task required during instrumental learning, while the task-irrelevant CSs were randomly presented in the background, one for each trial. The task was completely performed under extinction, so neither visual feedback nor aversive noises (USs) ever occurred. This phase lasted about 8 min and included 16 trials (4 for CS, of about 30 s each).

The rationale of this phase was to test direction and intensity biases respectively using the percentage of responses and grip force as dependent variables. Two different trial types were designed to disentangle the two biases.

Specifically, in the direction bias (or outcome-specific transfer) trials, participants were required to choose between R1 or R2 while CS+_1_ or CS+_2_ were presented. So, if the CS+_1_ was presented, choosing R1 would constitute a congruent response, while choosing R2 would constitute an incongruent response. Similarly, if the CS+_2_ was presented, choosing R1 would constitute an incongruent response, while choosing R2 would constitute a congruent response. Evidence for the direction bias would be seen if the presence of the CS induced a higher percentage of congruent responses, as compared to incongruent responses.

In the intensity bias (or general transfer) trials, participants were required to choose between R1 or R2 while CS+_3_ or CS− were presented. Importantly, none of the responses was compatible with the CS currently available (i.e., the responses were always associated with a different or no outcome). Evidence for the intensity bias would be seen if participants generally had greater grip force during the presentation of a CS+_3_, as compared to the CS−.

#### Skin conductance response (SCR) analysis

Galvanic skin conductance was recorded from electrodes (ambu WS) attached to subjects’ volar surface of the index and middle fingertip in their left hand and connected to a DC amplifier (Biopac Systems—MP150—GSR100). A gain factor of 5 µS/V and low-pass filter set at 10 Hz were used for recording the analog signal, which was then passed through the digital converter at a 200 Hz rate. The signal was then fed into AcqKnowledge 3.9 (Biopac Systems) and transformed into microsiemens for offline analysis. Skin Conductance Response (SCR) was extracted from the continuous signal. A SCR was considered valid if the trough-to-peak deflection started between 0.5–4.5 s following the CS onset, lasted for a maximum of 5 s, and was greater than 0.02 µS^75^. Smaller responses were encoded as zero. Raw SCR scores were square root transformed to normalize the distributions and scaled to each subject’s maximal response to the aversive stimulus, in order to account for inter-individual variability^75^.

#### Behavioral analysis

For the new analysis, we used the two indices of direction and intensity bias adopted in the original study. The direction bias index was computed as the difference between the percentage of congruent (i.e., consistent with the outcome predicted by the cue) and incongruent responses (i.e., not consistent with the outcome predicted by the cue). The intensity bias index was computed as the difference between the vigor (grip force) of the responses performed while presented with a punishment-associated conditioned stimulus (CS+) and with a stimulus associated with no punishment (CS−). For both indexes, the higher the value, the greater the bias (a value of 0 corresponded to the absence of the bias). We used the same method to detect outliers as in experiment 1, which revealed two outlier values. We performed the analysis including the outliers (although we obtained the exact same pattern of results with and without these outliers).

The two transfer indexes thus obtained were then statistically compared between males and females via null hypothesis significance testing (NHST) and Bayesian independent sample *t*-tests. The difference between the groups was quantified via estimation statistics^72,73^.

## Supplementary information

Supplementary materials

reporting-summary

## Data Availability

The full data set is available at the following URL: https://osf.io/te3hm/.

## Code availability

The scripts used for the analysis are available at the following URL: https://osf.io/te3hm/.
