# Supplementary material for: Sex differences in motivational biases over instrumental actions
Source: NPJ Sci Learn. 2024 Oct 8;9:62. doi: 10.1038/s41539-024-00246-6 (PMC11461879; doi:10.1038/s41539-024-00246-6)
Supplement: Supplementary file 1 — Supplementary materials [file 41539_2024_246_MOESM1_ESM.pdf]

SUPPLEMENTARY MATERIALS
Experiment 1
Explicit measures of liking and wanting

Following, we report in further detail the descriptive results of liking and wanting (pre- and post-Pavlovian learning phase) for the three selected outcomes (O_1, O_2, O_3) divided by sex (males; females) (see Supplementary table 1). We also report the descriptive results of liking/wanting differences between the three outcomes (see Supplementary table 2).

Supplementary table 1. Means (M), standard deviations (SD), minimal (Min) and maximal (Max) points of liking and wanting.

		**Liking pre**			
**Outcome**	**Sex**	**M**	**SD**	**Min**	**Max**
**O_1**	Males	7	1.62	4	9
**O_1**	Females	7.68	1.32	5	9
**O_2**	Males	7	1.97	1	9
**O_2**	Females	7.5	0.96	6	9
**O_3**	Males	7	1.52	4	9
**O_3**	Females	7.64	1.18	5	9
		**Liking post**			
**Outcome**	**Sex**	**M**	**SD**	**Min**	**Max**
**O_1**	Males	7.15	1.63	4	9
**O_1**	Females	7.81	1.03	6	9
**O_2**	Males	6.95	2.06	1	9
**O_2**	Females	7.57	1.03	6	9
**O_3**	Males	6.85	1.69	4	9
**O_3**	Females	7.9	1.14	5	9
		**Wanting pre**			
**Outcome**	**Sex**	**M**	**SD**	**Min**	**Max**
**O_1**	Males	6.45	1.73	3	9
**O_1**	Females	7.14	1.86	2	9
**O_2**	Males	6.6	2.09	1	9
**O_2**	Females	6.77	1.77	1	9
**O_3**	Males	6.45	1.32	4	9
**O_3**	Females	6.95	1.7	2	9
		**Wanting post**			
**Outcome**	**Sex**	**M**	**SD**	**Min**	**Max**
**O_1**	Males	7.2	1.77	3	9
**O_1**	Females	7.81	1.60	3	9
**O_2**	Males	7.15	2.11	2	9
**O_2**	Females	7.57	1.91	1	9
**O_3**	Males	7.4	1.5	3	9
**O_3**	Females	7.71	1.79	2	9

Supplementary table 2. Means (M), standard deviations (SD), minimal (Min) and maximal (Max) differences between outcomes in liking and wanting.

**Liking pre (differences)**

Sex	M	SD	Min	Max
**Males**	1.6	1.88	0	6
**Females**	1.36	0.73	0	3
		**Liking post (differences)**		
**Sex**	**M**	**SD**	**Min**	**Max**
**Males**	0.95	1.15	0	4
**Females**	0.9	1.04	0	4
		**Wanting pre (differences)**		
**Sex**	**M**	**SD**	**Min**	**Max**
**Males**	1.95	1.39	0	6
**Females**	1.36	1.18	0	4
		**Wanting post (differences)**		
**Sex**	**M**	**SD**	**Min**	**Max**
**Males**	1.45	1.39	0	6
**Females**	1.38	0.8	0	3

Transfer phase

Following, we report the analysis performed to test the direction bias using reaction times as dependent variable, and the two analyses performed to test the intensity bias using percentage of responses and response rate as dependent variables. Evidence for the direction bias would be seen if participants had faster reaction times while performing congruent responses, as compared to incongruent responses in the direction bias trials (i.e., respectively R_1 and R_2 while $CS+_1$ and $CS+_2$ were presented). Evidence for the intensity bias would be seen if the presence of the $CS+_3$ induced respectively a higher percentage of responses (R_1 and R_2), or a higher response rate (i.e., the average number of responses performed per trial) as compared to the CS- in the intensity bias trials. Three outlier values (one male and two females) were detected for the analysis of the percentage of responses. We performed the analysis including the outliers (although we obtained the exact same pattern of results with and without these outliers).

For the direction bias, we performed a mixed-measures ANOVA with response (Congruent/Incongruent) as within-subjects factor, sex (Males/Females) as between-subjects factor, and reaction times (Supplementary Figure 1A) as dependent variable. Three subjects were excluded from this analysis, since they had never chosen the incongruent response, so reaction times refer to a sample of 39 participants. Results showed no statistically significant effects, with Bayesian evidence in favor of the null hypothesis (response: $F_{1,37} = 0.06$; $p = 0.81$; $\eta_p^2 = 0.07$; $BF_{10} = 0.24$; sex: $F_{1,37} = 1.5$; $p = 0.23$; $\eta_p^2 = 0.04$; $BF_{10} = 0.66$; response by sex interaction: $F_{1,37} = 0.19$; $p = 0.89$; $\eta_p^2 = 0.001$; $BF_{10} = 0.24$). Overall, using reaction times as dependent variable, results did not show the presence of the direction bias or differences between sexes.

For the intensity bias, we performed two mixed-measures ANOVA with CS (CS+/CS-) as within-subjects factor, sex (Males/Females) as between-subjects factor, and, respectively, percentage of responses (Supplementary Figure 1B) and response rate (Supplementary Figure 1C) as dependent variables. Results showed no statistically significant effects, with Bayesian evidence in favor of the null hypothesis for both the percentage of responses (CS: $F_{1,40} = 2.73$; $p = 0.11$; $\eta_p^2 = 0.06$; $BF_{10} = 0.75$; sex: $F_{1,40} = 0.73$; $p = 0.4$; $\eta_p^2 = 0.02$; $BF_{10} = 0.66$; CS by sex interaction: $F_{1,40} = 0.96$; $p = 0.33$; $\eta_p^2 = 0.02$; $BF_{10} = 0.43$) and the response rate (CS: $F_{1,40} = 2.96$; $p = 0.09$; $\eta_p^2 = 0.07$; $BF_{10} = 0.84$; sex: $F_{1,40} = 0.45$; $p = 0.51$; $\eta_p^2 = 0.01$; $BF_{10} = 0.6$; CS by sex interaction: $F_{1,40} = 0.91$; $p = 0.35$; $\eta_p^2 = 0.02$; $BF_{10} =$

0.47). Overall, using percentage of responses or response rate as dependent variables, results did not show the presence of the intensity bias or differences between sexes.

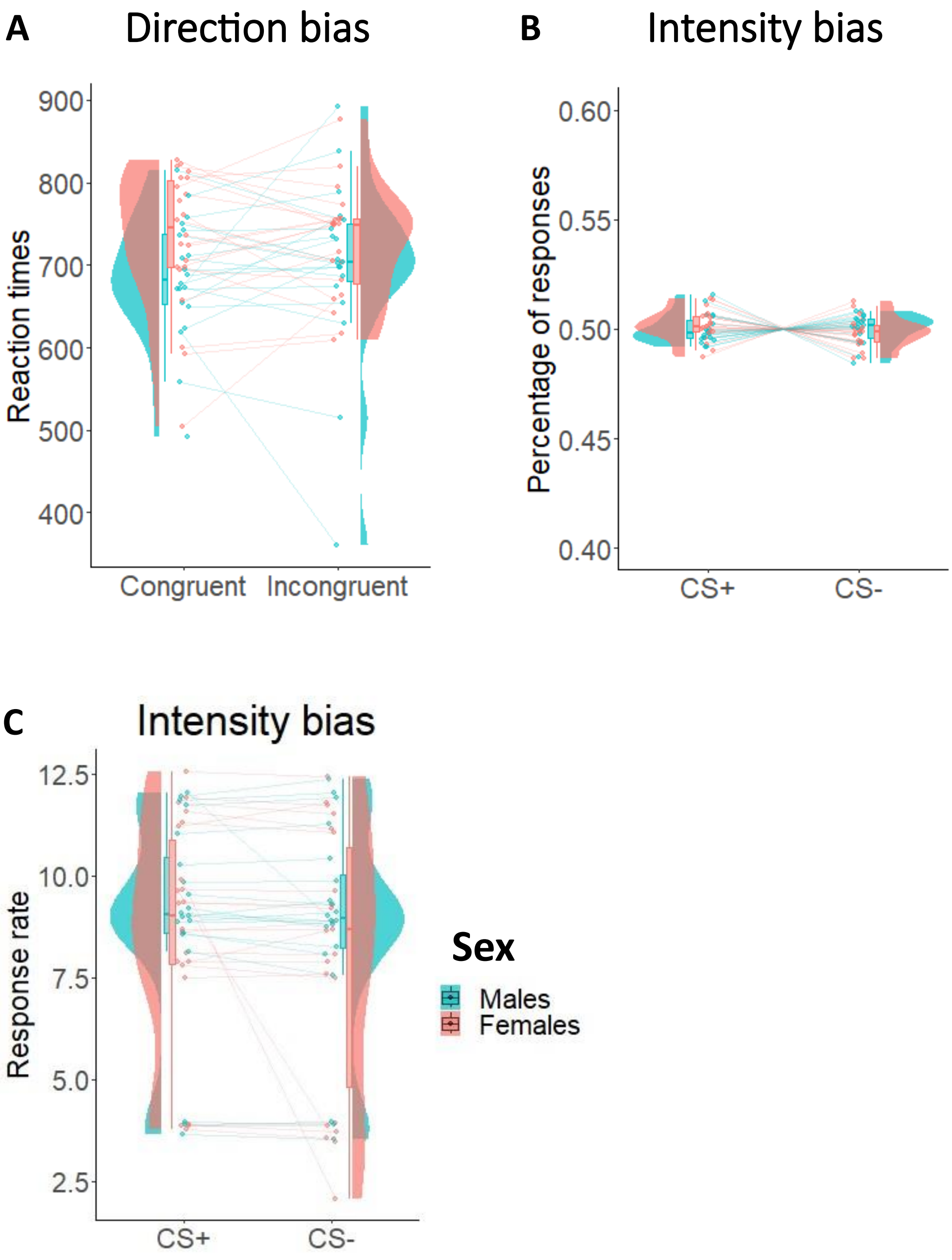

Supplementary Figure 1. Reaction times and percentage of responses across males and females in the transfer phase. The direction bias plot (A) shows the reaction times to congruent and incongruent responses in males and females. The intensity bias plots respectively show the percentage of responses (B) and the response rate (C) to CS+ and CS– in males and females. In all graphs, boxplots, individual scores, and data distributions are reported in coral for females and blue for males. Overall, data show no evidence for direction and intensity bias in both males and females using these dependent measures. For the first intensity bias plot (B), three detected outliers were excluded in order to enhance clarity, although the analysis included them.
