# Supplementary material for: Sex differences in motivational biases over instrumental actions
Source: NPJ Sci Learn. 2024 Oct 8;9:62. doi: 10.1038/s41539-024-00246-6 (PMC11461879; doi:10.1038/s41539-024-00246-6)
Supplement: Supplementary file 2 — reporting-summary [file 41539_2024_246_MOESM2_ESM.pdf]

nature portfolio

Corresponding author(s): Luigi Albert Enrico Degni

Last updated by author(s): Luigi A.E. Degni, Sara Garofalo, Gianluca FInc

Reporting Summary

Nature Portfolio wishes to improve the reproducibility of the work that we publish. This form provides structure for consistency and transparency in reporting. For further information on Nature Portfolio policies, see our Editorial Policies and the Editorial Policy Checklist.

Statistics

For all statistical analyses, confirm that the following items are present in the figure legend, table legend, main text, or Methods section.

n/a	Confirmed	
☐	☒	The exact sample size (*n*) for each experimental group/condition, given as a discrete number and unit of measurement
☐	☒	A statement on whether measurements were taken from distinct samples or whether the same sample was measured repeatedly
☐	☒	The statistical test(s) used AND whether they are one- or two-sided<br>*Only common tests should be described solely by name; describe more complex techniques in the Methods section.*
☐	☒	A description of all covariates tested
☐	☒	A description of any assumptions or corrections, such as tests of normality and adjustment for multiple comparisons
☐	☒	A full description of the statistical parameters including central tendency (e.g. means) or other basic estimates (e.g. regression coefficient) AND variation (e.g. standard deviation) or associated estimates of uncertainty (e.g. confidence intervals)
☐	☒	For null hypothesis testing, the test statistic (e.g. F, t, r) with confidence intervals, effect sizes, degrees of freedom and P value noted<br>*Give P values as exact values whenever suitable.*
☐	☒	For Bayesian analysis, information on the choice of priors and Markov chain Monte Carlo settings
☐	☒	For hierarchical and complex designs, identification of the appropriate level for tests and full reporting of outcomes
☐	☒	Estimates of effect sizes (e.g. Cohen's *d*, Pearson's *r*), indicating how they were calculated

Our web collection on statistics for biologists contains articles on many of the points above.

Software and code

Policy information about availability of computer code

Data collection	OpenSesame (version 3.2)
Data analysis	RStudio v1.0.136

For manuscripts utilizing custom algorithms or software that are central to the research but not yet described in published literature, software must be made available to editors and reviewers. We strongly encourage code deposition in a community repository (e.g. GitHub). See the Nature Portfolio guidelines for submitting code & software for further information.

Data

Policy information about availability of data

All manuscripts must include a data availability statement. This statement should provide the following information, where applicable:

- Accession codes, unique identifiers, or web links for publicly available datasets
- A description of any restrictions on data availability
- For clinical datasets or third party data, please ensure that the statement adheres to our policy

The full data set and scripts used for the analysis are available at the following URL: https://osf.io/te3hm/.

Research involving human participants, their data, or biological material

Policy information about studies with human participants or human data. See also policy information about sex, gender (identity/presentation), and sexual orientation and race, ethnicity and racism.

Reporting on sex and gender	In this study, we refer to "sex differences" because we categorized participants based on their sex, intended as a biological composition at birth, and not based on their gender identity. Specifically, participants were explicitly asked about their assigned sex at birth, rather than their gender identity.
Reporting on race, ethnicity, or other socially relevant groupings	*Please specify the socially constructed or socially relevant categorization variable(s) used in your manuscript and explain why they were used. Please note that such variables should not be used as proxies for other socially constructed/relevant variables (for example, race or ethnicity should not be used as a proxy for socioeconomic status).<br>Provide clear definitions of the relevant terms used, how they were provided (by the participants/respondents, the researchers, or third parties), and the method(s) used to classify people into the different categories (e.g. self-report, census or administrative data, social media data, etc.)<br>Please provide details about how you controlled for confounding variables in your analyses.*
Population characteristics	*Describe the covariate-relevant population characteristics of the human research participants (e.g. age, genotypic information, past and current diagnosis and treatment categories). If you filled out the behavioural & social sciences study design questions and have nothing to add here, write "See above."*
Recruitment	The recruitment process involved posting study announcements on relevant social networks groups and platforms. Interested individuals who responded to these posts were contacted for further screening and potential inclusion in the study. Specifically, potential participants were asked to report eventual history of neurological or psychiatric diseases, and if so, they were excluded from recruitment.
Ethics oversight	The study was conducted in accordance with institutional guidelines and the 1964 Declaration of Helsinki and was approved by the Bioethics Committee of the University of Bologna (Prot. 201070, 24/09/2020).

Note that full information on the approval of the study protocol must also be provided in the manuscript.

Field-specific reporting

Please select the one below that is the best fit for your research. If you are not sure, read the appropriate sections before making your selection.

☒ Life sciences ☐ Behavioural & social sciences ☐ Ecological, evolutionary & environmental sciences

For a reference copy of the document with all sections, see nature.com/documents/nr-reporting-summary-flat.pdf

Life sciences study design

All studies must disclose on these points even when the disclosure is negative.

Sample size	The number of participants was established based on a power analysis conducted on MorePower 6.0, for the planned 2×2 mixed-measures ANOVA, i.e., the design for both intensity bias and direction bias analyses. Specifically, the following parameters were used: Repeated Measures design factors = 1 factor (CS for intensity bias; Response for direction bias) with 2 levels (CS+, CS- for intensity bias; congruent, incongruent for direction bias); Independent Measures design factors = 1 factor (Sex) with 2 levels (Males, Females); Dependent Measures effect of interest = 1 factor with two levels; Independent Measures effect of interest = 1 factor with 2 levels; effect size (ηp2) = 0.17; significance level = 0.05; power = 0.8. The effect size was estimated based on the mean of effect sizes (ηp2) obtained by previous studies conducted with a similar task.
Data exclusions	Few subjects have been excluded by some supplementary analysis due to technical problems, but the main analyses involve the entire sample.
Replication	We performed two experiments; the second served as a control for the first and replicated the same results.
Randomization	Subjects were divided in males and females.
Blinding	See above.

Reporting for specific materials, systems and methods

We require information from authors about some types of materials, experimental systems and methods used in many studies. Here, indicate whether each material, system or method listed is relevant to your study. If you are not sure if a list item applies to your research, read the appropriate section before selecting a response.

Materials & experimental systems

n/a	Involved in the study
☒	☐ Antibodies
☒	☐ Eukaryotic cell lines
☒	☐ Palaeontology and archaeology
☒	☐ Animals and other organisms
☒	☐ Clinical data
☒	☐ Dual use research of concern
☒	☐ Plants

Methods

n/a	Involved in the study
☒	☐ ChIP-seq
☒	☐ Flow cytometry
☒	☐ MRI-based neuroimaging

Plants

Seed stocks

Report on the source of all seed stocks or other plant material used. If applicable, state the seed stock centre and catalogue number. If plant specimens were collected from the field, describe the collection location, date and sampling procedures.

Novel plant genotypes

Describe the methods by which all novel plant genotypes were produced. This includes those generated by transgenic approaches, gene editing, chemical/radiation-based mutagenesis and hybridization. For transgenic lines, describe the transformation method, the number of independent lines analyzed and the generation upon which experiments were performed. For gene-edited lines, describe the editor used, the endogenous sequence targeted for editing, the targeting guide RNA sequence (if applicable) and how the editor was applied.

Authentication

Describe any authentication procedures for each seed stock used or novel genotype generated. Describe any experiments used to assess the effect of a mutation and, where applicable, how potential secondary effects (e.g. second site T-DNA insertions, mosiacism, off-target gene editing) were examined.
